# Novel approach for genome scan meta-analysis of rheumatoid arthritis: a kernel-based estimation procedure

**DOI:** 10.1186/1753-6561-1-s1-s96

**Published:** 2007-12-18

**Authors:** Laurent Briollais, Gilles Durrieu, Ranodya Upathilake

**Affiliations:** 1 Samuel Lunenfeld Research Institute, Mount Sinai Hospital, 60 Murray Street, Toronto, Ontario MT5 3L9, Canada; 2 Public Health Sciences Department, University of Toronto, 155 College Street, Toronto, Ontario M5T 3M7, Canada; 3 Université Bordeaux 1 and Laboratoire GEMA, UMR CNRS 5805-EPROC, Place du Dr. Peyneau, Arcachon 33120, France

## Abstract

Genome scan meta-analysis (GSMA) can prove very useful in detecting genetic effects too small to be detected in an individual linkage study and can also lead to more consistent results. In this paper, we propose a new kernel-based estimation procedure for GSMA. Instead of estimating identity by descent between markers, as performed in interval mapping approaches, we estimated directly the nonparametric linkage score between markers using a kernel procedure. The GSMA is then extended to take into account the kernel estimate of the nonparametric linkage score and its variance at a given chromosomal position. The method is applied to the rheumatoid arthritis genome scan data (Genetic Analysis Workshop 15 Problem 2).

## Background

Rheumatoid arthritis (RA) is a chronic inflammatory disease that primarily affects the synovial tissues of multiple joints in the body. The etiology of the disease remains unknown, but it appears to have a complex genetic component. Several genome scans for RA studies have been performed to identify susceptibility loci, but most of the results have not been replicated [1]. These inconsistencies could arise from the small sample size, low statistical power, and clinical or genetic heterogeneity of these studies. Genome scan meta-analysis (GSMA) that combines the results from several linkage studies can have greater statistical power to detect small genetic effects and can lead to more consistent results. A general difficulty in GSMA is the heterogeneity across studies due to different marker maps, marker informativeness, sample sizes, sampling plans, and linkage tests. Loesgen et al. [2] proposed a meta-analytic test that computed a weighted average estimate of score statistics. Recently, Etzel et al. [3] used this method in a genome-wide meta-analysis of RA. Because of differences in marker maps across studies, they decided to align the marker maps after performing some interval mapping and combining nonparametric linkage (NPL) scores obtained from GeneHunter2 for markers in a pre-specified interval. Their method requires the estimation of identity-by-descent (IBD) sharing probabilities through the interval between two markers [4,5], which can be somewhat inaccurate and imprecise. The variability of the IBD estimate is difficult to measure and often not reflected in the GSMA. In this paper, we propose an alternative approach that estimates the NPL score between markers directly using a kernel-based estimation procedure. The GSMA is then extended to take into account the kernel estimate of the NPL score and its variance at a given chromosomal position.

## Methods

### Data

We have included three linkage studies in the meta-analysis of RA: NARAC (North American Rheumatoid Arthritis Consortium), ECRAF (European Consortium on Rheumatoid Arthritis Families) and United Kingdom (UK). Only microsatellite scans and RA affection status (binary outcome) were considered. NARAC has performed microsatellite scans for 511 mutliplex families, decomposed into 757 smaller families. ECRAF had high-density microsatellite data from 88 families, including 105 sib pairs typed with 1089 microsatellite markers. The UK group performed two screens: an initial screen of the entire genome using 369 markers analyzed on 175 families and a second screen performed on 197 families using 89 markers in regions showing evidence for linkage. The two screens were combined in our analyses. A summary of the data analyzed is given in Table 1.

**Table 1:** Summary of studies included in the meta-analysis

		No. of families			
			
Study	Population	Total	2 siblings	>2 siblings	No. of microsatellite markers
NARAC	U.S. Caucasian	757	208	535	396
ECRAF	French	88	16	72	1089
UK	U.K. Caucasian	372	158	213	369

### GSMA

We first performed a multipoint linkage analysis of each individual study and estimated the NPL score and marker information content (IC) at each marker location as well as at each systematic 2-cM interval using the program MERLIN [6]. We then performed the GSMA by calculating the weighted average of the NPL scores at a given chromosomal position [2]:

$$Z_{MA_{j}}=\frac{\sum_{i=1}^{k}w_{ij}Z_{ij}}{\sqrt{\sum_{i=1}^{k}w_{ij}^{2}}},$$

where *Z*_*ij *_is the NPL score from the *i*^th ^study at the *j*^th ^position, *k *is the number of studies, and *w*_*ij *_is the weight given to each study. To perform the GSMA, we used three different strategies that differ in the way the NPL score and IC are estimated between markers and the definition of the weight.

#### Method 1

Following Etzel et al. [3], the first method tries to align the marker maps. After the 2-cM interval mapping was completed with MERLIN, the NPL scores that were within 1 cM of each other were combined and the statistic ZMA was computed in each interval. The weight *w*_*ij *_is the product of the number of sib-pairs equivalents (SPE) from the *i*^th ^study and the IC estimated from MERLIN for the *i*^th ^study at the *j*^th ^interval:

*w*_*ij *_= *SPE*_i_*IC_*ij*_.

##### Method 2

This approach is not based on marker alignment. Instead of using an interval mapping estimate of the NPL score and IC between markers, we used a kernel regression method. The statistic ZMA is then computed at all marker positions available after merging the three data sets (i.e., if one marker is present in one study but missing in the other two, its associated NPL score and IC are estimated by the kernel regression). The weight is identical to Method 1 except that the IC is now replaced by its kernel estimate (ICK):

*w*_*ij *_= *SPE*_i_*ICK_*ij*_.

##### Method 3

The third approach is identical to Method 2 but now takes into account in the weight the precision of the kernel estimator, more precisely the inverse of standard deviation of NPL kernel estimator (*SDnpl*):

*w*_*ij *_= *SPE*_i_*ICK_ij_*I/*SDnpl*_*ij*_.

### Model

In Methods 2 and 3, the relationship between the NPL score (or the information content) (*Y*) and the marker location (*T*) is modelled using a nonparametric model given by:

*Y*_*i *_= *m*(*T*_*i*_) + *ε*_*i*_, for *i *= *l*,..., *n*,

where *m*(·) and *ε *denote, respectively, the regression function to be estimated and the model error; term *n *is the number of observations. The stochastic distribution *f*(·) of *ε *is typically unknown and is unlikely to follow any familiar distribution such as the normal distribution. Hence, we decided to use nonparametric statistics. The random variable enables to characterize the variation of *Y *around *m*(*t*), the mean regression curve with:

$$m(t)=E(Y/T=t)=\frac{\int yf(y,t)dy}{f\left(t\right)}.$$

So, the regression function *m*(·) depends on the joint and the marginal densities, which are both unknown. A density estimator allows the analysis of data sets that could exhibit skewness and multimodality due to different factors (for example, mixture of several distributions and clusters). A histogram type estimator is the most frequently used but could be strongly affected by the choice and number of classes chosen. So we decided to use a nonparametric kernel estimator that behaves much better statistically. A kernel estimator of a function *f*(·) is defined by:

$${\hat{f}}_{h}(t)=\frac{1}{nh}\sum_{i=1}^{n}K\left(\frac{t-T_{i}}{h}\right),$$

where *n *is the sample size, *h *is the bandwidth (the smoothing parameter) to be determined, and *K *is the continuous fixed kernel function with finite variance generally satisfying *K *> 0, *K*(-t) = *K*(t), and ∫ *K*(*t*) *dt *= 1. Here we considered the Gaussian kernel. This raises the question of determining the bandwidth parameter. The estimator ${\hat{f}}_{h}(.)$ is wiggly when *h *is small and very flat when *h *is large. Different procedures have been previously proposed to determine *h*, for example cross-validation. The problem in our application is that we need to estimate the NPL score function at different marker locations using the kernel procedure but also its variance. Both the kernel estimator and the variance depend on the same smoothing parameter and an optimal choice for the kernel estimator might not be optimal for the variance. To our knowledge, there is no optimal procedure for this problem. For that reason, we could not apply the classical cross-validation procedure, so we decided to choose the bandwidth empirically. The bandwidth was chosen inversely proportional to the number of markers of each individual study on each chromosome. Therefore, *h *was not constant in our study but depended on the genetic background. More exactly, we chose: $h=cst*\sum_{i=1..k}M_{i}/M_{i}$, where *M*_*i *_is the number of markers of each individual linkage study on one particular chromosome and the constant was fixed to 4.0. Intuitively, we understand that a study with less markers yields more variable results. Applying a larger *h *leads to a smoother function and thus to a decreased variability. This determination of *h *provided a good estimation of both the NPL score function and its variance.

Using the kernel estimator of the marginal and joint densities function, the regression estimator of *m*(·) is:

$${\hat{m}}_{h}(t)=\frac{\sum_{i=1}^{n}K_{h}(t-T_{i})Y_{i}}{\sum_{i=1}^{n}K_{h}(t-T_{i})},$$

where *K*_*h*_(·) was chosen to be the Gaussian kernel function here. This form of the estimate of the regression curve was proposed by Nadaraya [7] and Watson [8]. The estimator ${\hat{m}}_{h}(.)$ is a consistent estimator *m*(*t*) and normally distributed when *h *tends toward 0. It is also shown [9,10] that under regularity conditions when *h *tends toward 0, the variance of the estimator ${\hat{m}}_{h}(.)$ can be approximated by:

$$\hat{V}ar\left[{\hat{m}}_{h}(t)\right]\approx \frac{1}{nh}\frac{\sigma^{2}(t)}{f(t)}{\left\|K\right\|}_{2}^{2},\text{ where }{\left\|K\right\|}_{2}^{2}=\int K^{2}(t)dt$$

and ${\hat{\sigma }}^{2}(t)=\frac{1}{n}\sum_{i=1}^{n}\frac{K_{h}(t-T_{1})}{{\hat{f}}_{h}\left(t\right)}{\left(Y_{i}-{\hat{m}}_{h}(t)\right)}^{2}$ is the estimator of ${\hat{\sigma }}^{2}$(*t*).

In our method 3 above, we took $SDnpl=\sqrt{Var\left[{\hat{m}}_{h}(t)\right]}$. All our computations were preformed with the computer program *R *for Linux.

## Results

**Figure 1:**
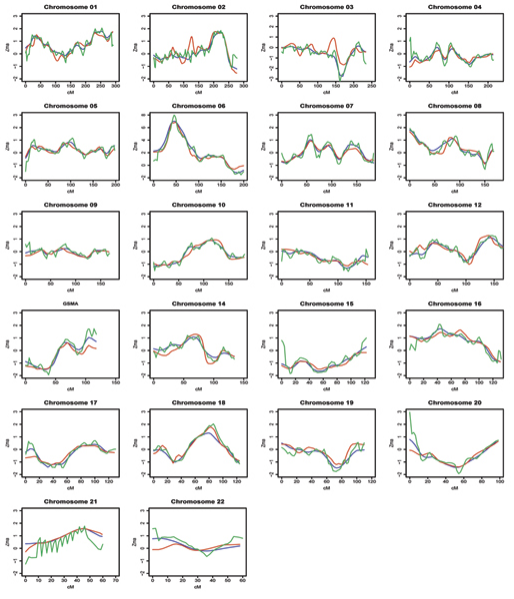
**GSMA results on the 22 autosomes**. Green line, method 1; blue line, method 2; red line, method 3.

**Figure 2:**
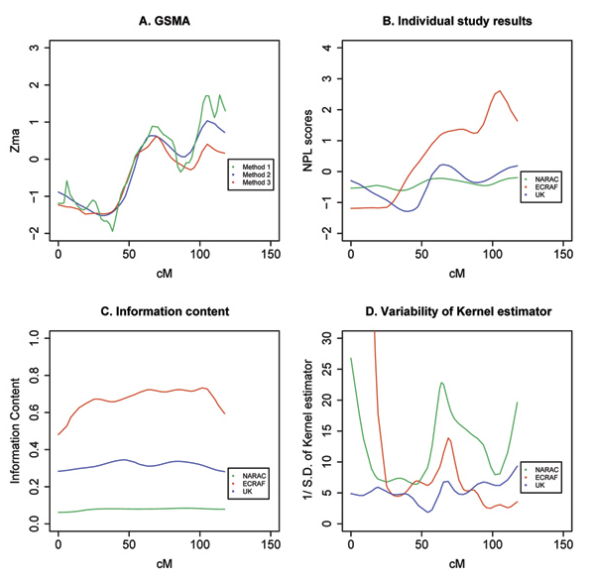
GSMA results on chromosome 13.

The results of the *Z_MA _*test statistic on the whole genome are presented in Figure 1. Our results confirm the role of the HLA region in the susceptibility to RA with a *Z_MA _*test statistic close to 8.0 on chromosome 6 (Fig. 1). The three methods gave consistent results for this chromosome. We also found some suggestion of linkage on chromosomes 1 (240 to 260 cM), 2 (200 to 250 cM), 8 (10 cM), 16 (40 cM), 18 (80 to 90 cM), and 21 (45 cM), with a *Z_MA_* test statistic close to 2.0. For most chromosomes, the three methods gave very similar results. Method 2 performs identically to Method 1, and this was expected because the kernel estimation is only used to smooth the information content and NPL score functions. Some differences, however, were observed between Method 3 and the two others, especially on chromosomes 2, 3, 13, 21, and 22. To better understand these discrepancies, we describe the GSMA results on chromosome 13, where Methods 1 and 2 gave a larger *Z_MA_* test statistic than Method 3 at the location 120 cM (Fig. 2A). The linkage signal was stronger in ECRAF (NPL score > 2.0) than in NARAC and UK studies (NPL score close to 0) (Fig. 2B) and the information content of ECRAF was also larger (Fig. 2C).

However the variance of the NPL score at this location, as estimated by the kernel regression procedure, was higher for ECRAF than for the two other studies (Fig. 2D). Method 3, unlike Methods 1 and 2, weights each study inversely proportionally to this variance and therefore led to a lower *Z_MA_* test statistic. Moreover, the peak of linkage in ECRAF is relatively thin, which could be associated with a larger variance of the kernel estimator at this location (Fig. 2B). This is because the variance of the kernel estimator is inversely proportional to the density estimate of the NPL score at one particular location (see variance formula above). In general, denser marker regions and wider peak regions both could contribute to a low variance of the kernel estimator and hence, to a larger GSMA statistic.

## Discussion

The use of kernel-based regression methods allow us to estimate the NPL score function at various locations along the genome and thus make possible the meta-analysis of several linkage studies with different genetic maps. To our knowledge, this is the first kernel-based approach for GSMA studies. Previous GSMAs have tried to perform some map alignment that requires an estimation of the IBD sharing probabilities between markers using interval mapping. However, the variability of this estimate is not reflected in the GSMA statistic. An important advantage of our approach is that it is completely nonparametric and we can obtain a measure of the variability of the NPL score estimate along the genome. Incorporating this variability into the GSMA statistic (Method 3) might improve the consistency of linkage results by over-weighting studies with more precise estimate of the NPL score function. This could reflect, for example, a higher marker density. A larger weight will be given to a study that finds a linkage peak with many markers than to a study that finds the same peak with fewer markers. Therefore, the information about NPL score variability is very useful to weight each individual study. Our procedure can also down-weight thin peaks. Further simulation studies are needed to better understand its properties, in particular in terms of detection of true linkage peaks.

## Competing interests

The author(s) declare that they have no competing interests.
